# Antibody-Based Therapies in Multiple Myeloma

**DOI:** 10.1155/2011/924058

**Published:** 2011-03-02

**Authors:** Yu-Tzu Tai, Kenneth C. Anderson

**Affiliations:** Department of Medical Oncology, Jerome Lipper Multiple Myeloma Center, Dana-Farber Cancer Institute, Harvard Medical School, 44 Binney Street, Boston, MA 02115, USA

## Abstract

The unmet need for improved multiple myeloma (MM) therapy has stimulated clinical development of monoclonal antibodies (mAbs) targeting either MM cells or cells of the bone marrow (BM) microenvironment. In contrast to small-molecule inhibitors, therapeutic mAbs present the potential to specifically target tumor cells and directly induce an immune response to lyse tumor cells. Unique immune-effector mechanisms are only triggered by therapeutic mAbs but not by small molecule targeting agents. Although therapeutic murine mAbs or chimeric mAbs can cause immunogenicity, the advancement of genetic recombination for humanizing rodent mAbs has allowed large-scale production and designation of mAbs with better affinities, efficient selection, decreasing immunogenicity, and improved effector functions. These advancements of antibody engineering technologies have largely overcome the critical obstacle of antibody immunogenicity and enabled the development and subsequent Food and Drug Administration (FDA) approval of therapeutic Abs for cancer and other diseases.

## 1. Introduction

Despite the landmark approval of the anti-CD20 mAb rituximab for the treatment of B-cell malignancies, to date, no mAb-based therapy has been approved for MM treatment. The development of effective cytotoxic mAb therapies in MM has been hindered by the lack of uniquely and constitutively expressed target molecules on all MM cells. Indeed, studies in early 2000 demonstrated only minimal activity of anti-CD20 rituximab and antibodies against plasma cell-specific CD38 antibodies in MM [1–4]. However, numerous efforts to identify new targets on MM cells including gene expression profiling and oncogenomic studies are under way. Derived mAbs (e.g., against CD40, HM1.24, IGF-1R, CD56, CS1, CD138, CD74, IL-6R, CD38, TRAIL-R1, and the activin receptor type IIA (ActRIIA)) have already demonstrated promising preclinical as well as early clinical activity (Table 1). 

Given the importance of the bone marrow (BM) microenvironment for MM cell growth, survival, and drug resistance, mAbs have been additionally designed to functionally block both autocrine and paracrine secreted cytokines and growth factors as well as molecules mediating MM-stromal cell interaction. For example, mAbs targeting interleukin-6 (IL-6), vascular endothelial growth factor (VEGF), Receptor Activator of NF*κ*B ligand (RANKL) (also known as osteoprotegerin ligand (OPGL)), and Dickkopf homolog 1 (DKK1) are among those under clinical evaluation. Specifically, targeting bone-MM cell interactions *via* bone biology modulating factors such as DKK1 and RANKL is likely to trigger anti-MM effects but also improves bone disease thereby improving both patient survival as well as patient's quality of life.

In the coming years, the preclinical progress in defining novel MM markers will be continued and subsequently will advance the clinical development of therapeutic mAbs, alone or in combination with other anti-MM agents, to improve patient outcome in MM.

## 2. Mechanisms of Action of Therapeutic Monoclonal Antibodies

**Figure 1:**
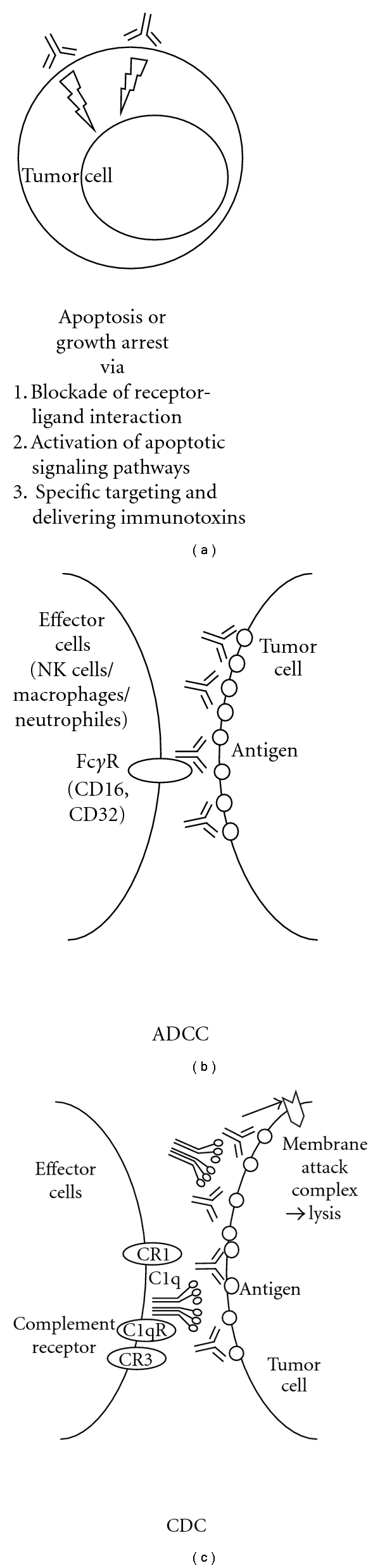
Mechanisms of actions associated with therapeutic monoclonal antibodies. (a) Therapeutic antibodies could directly induce apoptosis or growth arrest upon binding to cell surface antigen on tumor cells. Rituximab and Mapatumumab (anti-TRAIL-R1) could induce growth inhibition or apoptosis signaling to directly block tumor cell growth and survival. Such mechanism of action was employed by mAbs conjugated with toxins, that is, maytansinoids (DM1, DM4) for BB-10901 (anti-CD56) and BT062 (anti-CD138), thus directly target and eliminate tumor cells. Most of the approved therapeutic mAbs belong to IgG1 subclass, which has a long half-life and trigger potent immune-effector functions. (b) Following the binding of mAbs to a specific target on a tumor cells, antibody-dependent cellular cytotoxicity (ADCC) is triggered by interactions between the Fc region of an antibody bound to a tumor cell and Fc receptors, particularly FcRI and FcRIII, on immune effector cells such as neutrophils, macrophages, and natural killer cells. MAb-coated tumor cells are phagocytosed by macrophages or undergo cytolysis by NK cells. (c) In the case of complement-dependent cytotoxicity (CDC), recruitment of C1q by IgG bound to the tumor cell surface is an obligatory first step. This triggers a proteolytic cascade that leads to generation of the effector molecule, C3b, and then to formation of a membrane attack complex that kills the target cell by disrupting its cell membrane.

Antibodies of IgG, the most commonly used immunoglobulin form in cancer therapy, are unique proteins with dual functionality. Therapeutic mAbs use one or more following mechanisms (Figure 1) to reduce tumor burden in patients. They can be categorized into direct and indirect actions. Three modes of action could be further subcategorized from the direct action (Figure 1(a)) of mAb-based cancer therapy, including blocking the function of target signaling molecules or receptors, stimulating apoptosis signaling cascades, and targeting function to selectively target tumor cells and deliver toxins. The receptor functional blocking can occur by inhibiting ligand binding to inhibit cell cycle progression, DNA repair, or angiogenesis. It could also occur by increasing internalization of receptors or decreasing proteolytic cleavage of receptors. In the case of targeting function, mAbs could be conjugated with immunotoxins, that is, antitubulin agents (DM1/DM4, auristatin), doxorubicin, radioisotopes, or other chemotherapeutic drugs, thus selectively targeting and killing tumor cells. Indirect action of mAb therapy is mediated by the immune system. The elimination of tumor cells using mAbs depends on Ig-mediated mechanisms, including antibody-dependent cellular cytotoxicity (ADCC) and complement-dependent cytotoxicity (CDC), to activate immune effector cells to lyse target tumor cells (Figure 1(b)) These two mechanisms are believed to have the greatest impact, although there are conflicting views of which of these two pathways contributes the most to the response. ADCC involves the recognition of the Ab by immune cells that engage the Ab-marked cells and either through their direct action, or through the recruitment of other cell types, led to the tagged-cell's death. CDC (Figure 1(c)) is a process where a cascade of different complement proteins become activated, usually when several IgGs are in close proximity to each other, either with one direct outcome being cell lysis, or one indirect outcome being attracting other immune cells to this location for effector cell function.

## 3. Antibodies Targeting Cell Surface Protein on MM Cells

Several mAbs directed against MM cell surface are being investigated as potential therapy in MM. Listed below are mAbs against receptor antigens that are currently under clinical development or investigation in MM.

### 3.1. Limited Clinical Benefit from Anti-CD20 mAb Rituximab in MM

MM is usually not considered as a disease suitable for anti-CD20 therapy due to weak CD20 expression in the majority of patients. For example, results from a clinical phase II trial in relapsed MM showed that Rituximab treatment yielded significant reductions in circulating B cells and serum IgM levels but had no beneficial clinical effect [5]. 

Moreover, rituximab was investigated for maintenance therapy in MM following autologous hematopoietic stem cell transplantation (SCT) [2]. Although the number of MM patients was too low to draw definitive conclusions, the use of rituximab in this setting was associated with an unexpectedly high rate of early relapse. The authors therefore hypothesized a possible role for rituximab in provoking a further decrease in the residual, normal B-cell activity within the context of the complex network of antitumor immune response. Taken together, the resistance of MM cells against rituximab could be due to the level of CD20 expression, dissociated action of CDC and ADCC, polymorphism in FGCR3 (CD16) receptor, and an inadequate dose schedule.

 In contrast, other studies demonstrated that the CD20+ phenotype is associated with patients with t(11,14)(q13;q32) and with shorter survival [6] and that occasional clinical responses have been achieved in selected patients with CD20^+^ myelomatous plasma cells [7, 8].

Finally, new insights suggest that circulating CD20^+^ clonotypic B cells act as precursors or “neoplastic stem cells” in MM patients, representing the proliferative compartment of the disease able to play a role in determining relapse after effective treatments [9]. Thus, clinical trials using rituximab in MM may deserve further investigation.

### 3.2. Monoclonal Antibodies Targeting IL-6R to Overt IL-6/IL-6R Function

IL-6 is a major growth and survival factor in MM cells whose effects are mainly paracrine [10]. Various therapeutic agents which affect IL-6-mediated effects have been tested including IL-6-conjugated mAbs directed against IL-6R and IL-6 [11]. IL-6R antagonist SANT-7, in combination with Dex and all-transretinoic acid (ATRA) or zoledronic acid, strongly inhibited growth and induced apoptosis in MM cells [12–14]. These studies suggest that overcoming IL-6-mediated cell resistance by SANT-7 potentiates the effect of glucocorticoides and bisphosphonates on MM cell growth and survival, providing a rationale for therapies using IL-6 antagonists in MM. 

Tocilizumab (MRA, atlizumab, Roche Pharmaceuticals) is a humanized anti-human IL-6R mAb (rhPM-1, IgG1 class) designed by using genetic engineering technology and the first therapeutic mAb developed in Japan [15]. Tocilizumab specifically blocks IL-6 actions and ameliorates diseases associated with IL-6 overproduction [16]. For example, besides Castleman's disease and rheumatoid arthritis (RA), tocilizumab has been shown to be effective in patients with juvenile idiopathic arthritis and Crohn's disease [17, 18]. Tocilizumab treatment is generally well tolerated and safe. Moreover, blockade of IL-6R may prove effective in limiting MM cell growth. Indeed it is now evaluated in open-label Phase I (USA) and II (France) trials to assess its safety and efficacy as monotherapy in MM patients who are not candidates for, or who have relapsed after stem cell transplantation (SCT).

In addition, NRI, another receptor inhibitor of IL-6 genetically engineered from tocilizumab, is under preclinical evaluation [19]. NRI consists of VH and VL of tocilizumab in a single-chain fragment format dimerized by fusing to the Fc portion of human immunoglobulin G1. The binding activity to IL-6R and the biological activity of the purified NRI were found to be similar to those of parental tocilizumab. Because NRI is encoded on a single gene, it is easily applicable to a gene delivery system using virus vehicles. An adenovirus vector encoding NRI was administered to mice intraperitoneally (i.p.) and monitored for the serum NRI level and growth reduction property on the xenografted IL-6-dependent MM cell line S6B45. These findings indicate that NRI is a promising agent applicable to the therapeutic gene delivery approach for IL-6-driven diseases.

### 3.3. Targeting CD40 by SGN-40 or HCD122

Novel monoclonal antibodies targeting CD40 activation in MM cells, SGN-40/Dacetuzumab (Seattle Genetics, Genentech) and HCD122/Lucatumumab (Novartis), have been investigated [20, 21]. In preclinical studies, SGN-40, a humanized IgG_1_ partial agonistic mAb mediates cytotoxicity against CD40-expressing MM cell lines and patient MM cells via suppression of IL-6-induced proliferative and antiapoptotic effects, as well as ADCC [20]. SGN-40 also induced significant antitumor activity in xenograft mouse models of human MM and lymphoma [22]. HCD122 (CHIR12.12) (Novartis), a novel, fully human, IgG_1_ antagonistic mAb specifically blocked CD40L-induced adhesion, cytokine secretion, and survival of MM, as well as induced marked ADCC against CD40+ MM cells [21]. *In vivo* anti-MM activity by HCD122 was demonstrated in a xenograft model of 12BM MM plasmacytoma in mice [23]. Early clinical trials have evaluated the pharmacokinetics, safety, and efficacy of dacetuzumab monotherapy in patients with relapsed/refractory MM and other B-cell tumors [24, 25]. Phase I data suggest both agents are well tolerated with no immunogenicity and show early evidence of single-agent clinical activity in relapsed and refractory MM and NHL [26, 27]. SGN-40 Phase Ib clinical trials in combination with lenalidomide and dexamethasone/or bortezomib are planned based on enhanced anti-MM activities when combining SGN-40 with lenalidomide [28].

### 3.4. Targeting CS1 by HuLuc63/Elotuzumab in MM

Using subtractive hybridization of naïve B-cell cDNA from memory B/plasma cell cDNA, CS1 (CD2 subset-1, CRACC, SLAMF7, CD319), a novel member of the signaling lymphocyte activating molecule- (SLAM-) related receptor family, was identified to be highly expressed in plasma cells [29, 30]. Specifically, CS1 mRNA and protein are expressed in CD138-purified primary tumor cells from the majority of MM patients (>97%), but neither in major body organs nor CD34+ stem cells. To a low extent, its expression was also observed in NK cells, a subset of T cells, activated monocytes and activated dendritic cells. CS1 may contribute to MM pathogenesis by increasing MM cell adhesion, clonogenic growth, and tumorigenicity via c-maf-mediated interactions with BMSCs [31]. A novel humanized anti-CS1 mAb HuLuc63 (elotuzumab) was selected for clinical development due to its potent tumor-killing activity *in vivo* and *in vitro*. Specifically, elotuzumab induced significant ADCC against MM cells even in the presence of BMSCs. Moreover, it triggered autologous ADCC against primary MM cells resistant to conventional or novel therapies including bortezomib and HSP90 inhibitor, and markedly enhanced HuLuc63-induced MM cell lysis when pretreated with conventional or novel anti-MM drugs [29, 32]. 

A phase I study of HuLuc63 was well tolerated in MM patients [33]. Preliminary PK data reveal that peak serum drug levels for the 0.5 mg/kg dosing cohort reached 10 mg/mL, which was sufficient to achieve CS1 saturation of at least 70% on the antigen rich NK cell subset. Drug levels dropped below 1 mg/mL by day 7, however, coinciding with a decrease in saturation. This indicates that the higher doses to be used in subsequent cohorts may achieve and surpass sustained concentrations in patients above this level. Enrollment is continuing to determine the MTD. Early results of clinical trials of HuLuc63 in combination with bortezomib or lenalidomide or dexamethasone were reported at the ASH meeting 2009 [34, 35], suggesting that elotuzumab may enhance the activity of bortezomib and lenalidomide in treating MM with acceptable toxicity. PK analysis suggests a serum half-life of 10-11 days at higher doses (10 and 20 mg/kg). Preliminary analysis of mononuclear cells of peripheral blood and the BM indicates that objective responses correlate well with complete saturation of CS1 sites by elotuzumab on BM plasma cells and NK cells. The combination of elotuzumab with lenalidomide and low-dose dexamethasone has a manageable adverse event profile and compared to historical data for lenalidomide and high-dose dexamethasone, the preliminary efficacy data (PR of 92%) are very encouraging.

### 3.5. Targeting CD56 with Immunotoxin-Conjugated mAb

HuN901 conjugated with the maytansinoid N^2′^-deacetyl-N^2′^-(3-mercapto-1-oxopropyl)-maytansine (DM1), a potent antimicrotubular cytotoxic agent may provide targeted delivery of the drug to CD56-expressing tumors including MM. HuN901-DM1 has significant *in vitro* and *in vivo* anti-MM activity at doses that were well tolerated in a murine model [36].

The phase I clinical study of huN901-DM1 (BB-10901) in 23 MM patients determined the MTD as 140 mg/m^2^/week dose and demonstrated an overall favorable safety profile [37]. Exciting single agent activity was observed in heavily pretreated MM patients, which warrant continued investigation of this novel agent in MM patients especially when used in combination with approved anti-MM agents/regimens such as lenalidomide and dexamethasone.

### 3.6. Targeting CD38 in Multiple Myeloma

The CD38 molecule is expressed on cell surfaces in a majority of lymphoid tumors, notably MM [38, 39]. However, early studies using anti-CD38 mAb with or without an immunotoxin (ricin) have not led to useful clinical applications [4, 40].

Recently, a human anti-CD38 IgG_1_ HuMax-CD38 (Daratumumab) was raised after immunizing transgenic mice (HuMax-Mouse) possessing human, but not mouse, Ig genes. Preclinical studies indicated that HuMax-CD38 was effective in killing primary CD38+CD138+ patient MM cells and a range of MM/lymphoid cell lines by both ADCC and CDC [41]. In SCID mouse animal models, using sensitive bioluminescence imaging, treatment with Hu.

Max-CD38 inhibited CD38+ tumor cell growth in both preventive and therapeutic settings. In addition, HuMax-CD38 inhibits the CD38 ADP-ribosyl cyclase activity in target cells, which may contribute to the effectiveness of HuMax-CD38 in killing both primary MM and plasma cell leukemia cells.

Similarly, MOR202 (MorphoSysAG), a fully human anti-CD38 IgG_1_ mAb produced by a human combinatorial antibody library (HuCAL) platform, also efficiently triggers ADCC against CD38+ MM cell lines and patient MM cells *in vitro* as well as *in vivo* in a xenograft mouse model [39, 42]. One practical problem in applying anti-CD38 therapy is the wide expression on lymphoid, myeloid, and epithelial cells, especially following cell activation. However, mAbs specifically blocking CD38 might still provide a new approach for interfering with deleterious growth circuits, therefore increasing the susceptibility of MM and leukemic cells to conventional chemotherapy.

### 3.7. Targeting HM1.24 on MM Cells

HM1.24 (CD317) was originally identified as a cell surface protein differentially overexpressed on MM cells [43] and later was found to be identical to bone stromal cell antigen 2 (BST-2). A role of HM1.24 in trafficking and signaling between the intracellular and cell surface of MM cells was suggested since it is one of the important activators of NF-kappaB pathway [44]. The humanized anti-HM1.24 mAb (IgG1/kappa, AHM, Chugai Pharmaceutical Co., Ltd.) is able to effectively induce ADCC against some human myeloma cells in the presence of human PBMCs as effectively as a chimeric anti-HM1.24 mAb [45]. Single intravenous injection of AHM significantly inhibited tumor growth in both orthotopic and ectopic human MM xenograft models [46]. Although limited, the only one phase I/II clinical study reported that a humanized anti-HM1.24 mAb did not cause any serious toxicity when administered to patients with relapsed or refractory MM [46].

Most recently, we characterized XmAb5592, a novel Fc-engineered and humanized anti-HM1.24 mAb, and studied mechanisms of its anti-MM activity [47]. XmAb5592, with double amino acid substitution in Fc region of the wild-type IgG1, has approximately 40-fold and 10-fold increases in affinity for Fc gamma receptor III (FcRIIIa) and (FcRIIa), respectively, expressed on effector cells including NK cells. It triggers 10–100-fold higher ADCC against these MM cell lines than a native/non-Fc-engineered version (anti-HM1.24 IgG1) of the Ab. XmAb5592 also induced more potent anti-MM activity in murine subcutaneous xenograft murine models using RPMI 8226 cells. These results suggest that XmAb5592 is a promising next generation immunotherapeutic for MM.

### 3.8. Targeting TRAIL Death Signaling Pathway

Two human agonistic mAbs directed against TRAILR1 (HGS-ETR1, TRM-1, Mapatumumab) and TRAILR2 (HGS-ETR2) killed 68% and 45% of MM cell lines, respectively, [48]. Only 18% of MM cell lines are resistant to either antibody. There is no correlation between TRAILR expression level and sensitivity to TRAIL-R1 or TRAIL-R2 triggering. Both the extrinsic (caspase 8, Bid) and the intrinsic (caspase 9) pathways are activated by anti-TRAIL mAbs. Mapatumumab is well tolerated in a phase I study in patients with advanced solid malignancies (*n* = 41) and 12 patients had stable disease for 1.9 to 29.4 months [49]. These studies encouraged clinical trials of anti-TRAILR1 mAb in MM. In addition, based on enhanced cytotoxicity when combining mapatumumab with bortezomib in preclinical experiments [50], a randomized phase II study was recently started comparing TRM-1 plus bortezomib (Velcade) versus bortezomib alone in patients with relapsed or refractory MM.

### 3.9. Targeting CD74 with Milatuzumab

CD74 is an integral membrane protein that functions as a MHC class II chaperone. Milatuzumab is a humanized anti-CD74 mAb constructed using the same human backbone as epratuzumab (anti-CD22), whose safety has been demonstrated in clinical trials of patients with B-cell malignancies and autoimmune disorders [51, 52]. MM cell lines express CD74 (~60% of samples) and milatuzumab caused growth inhibition and induction of apoptosis in CD74-expressing MM cell lines when cross-linked with an anti-human immunoglobulin G secondary antibody [53]. Moreover milatuzumab demonstrated promising therapeutic activity in a CAG-SCID mouse model of disseminated disease for MM when used alone or in combination with doxorubicin, dexamethasone, bortezomib, or lenalidomide [54, 55]. In a phase I trial, milatuzumab showed no severe adverse effects in patients with relapsed/refractory MM, and it stabilized the disease in some patients for up to 12 weeks [51]. Supporting the data in MM ongoing clinical trials testing different treatment schedules of milatuzumab in chronic lymphocytic leukemia, non-Hodgkin's lymphoma, and MM indicate that milatuzumab shows no severe adverse effects in humans.

## 4. Antibodies Targeting MM Cells in the Bone Marrow Microenvironment

MM cells are highly dependent on the BM microenvironment for growth and survival through interactions particularly with BM stromal cells (BMSCs) and osteoclasts, which secrete important MM growth factors and cytokines. Importantly, these factors/cytokines are further induced from BMSCs when MM cells adhere to BMSCs [10]. Thus, mAbs designed to block the binding of MM cell growth and survival factors to their cognate receptors have been under intensive development.

### 4.1. Blockage of IL-6 Binding to MM Cells

Early work in developing mAb-based immunotherapies for MM has been focused on the blockade of IL-6 secretion from BM microenvironment because of its key role in promoting MM cell growth and survival. Initial studies of mouse mAb to IL-6 (murine BE-4 and BE-8) demonstrated a transient tumor cytostasis and reduction in toxicities from IL-6 [56]. The potential of combination therapy, including BE-8 (250 mg), Dex (49 mg/day), and high-dose melphalan (220 mg/m^2^ (HDM220)), followed by autologous SCT was demonstrated for the treatment of 16 patients with advanced MM. Overall, 13 of 16 patients (81.3%) exhibited a response, with a complete response (CR) seen in 6 patients (37.5%) without any toxic or allergic reactions. However, the incidence of thrombocytopenia and neutropenia increased. Subsequent clinical trials of BE-8 concluded that limitations of this regimen are first the amount of BE-8 that can be injected due to its short half-life (3-4 days) and second, the continued production of IL-6 *in vivo*. Most recently, a high-affinity fully human version of BE-8, OP-R003-1 (or 1339, Azintrel), was selected through ActivMAb antibody discovery technology. Indeed, it enhanced cytotoxicity induced by dexamethasone, as well as bortezomib, lenalidomide, and perifosine, in a synergistic fashion [57]. Importantly, Azintrel also blocked bone turnover in SCID-hu mouse model of MM, providing an additional rationale for its use in MM. 

Despite overcoming the safety concerns of human anti-mouse antibodies associated with murine anti-IL-6 mAb and a long half-life (17.8 days) in circulation, the chimeric mouse mAb to IL-6 CNTO 328 has been ineffective in producing a meaningful response in MM [58, 59]. Nevertheless, due to enhanced anti-MM activities of combined CNTO 328 and bortezomib/or dexamethasone in preclinical models, ongoing studies are investigating these regimens for their clinical value in treating MM [60, 61]. Specifically, results of a small safety analysis (*n* = 21) done as a run-in to a larger ongoing Phase II trial showed promising preliminary efficacy of CNTO 328 in combination with bortezomib in relapsed/refractory MM. CNTO 328 is also being evaluated as part of a combination therapy for initial treatment of MM in a Phase II trial which compares the safety and effectiveness of CNTO 328 plus Velcade-melphalan-prednisone (VMP) with VMP alone.

### 4.2. Targeting MM-Induced Bone Lesion

#### 4.2.1. Targeting RANK/RANKL/OPG Axis Using Denosumab for MM-Associated Bone Destruction

Receptor activator of nuclear factor-kappaB ligand (RANKL) is a cytokine member of the tumor necrosis factor family that is the principal mediator of osteoclastic bone resorption [62]. Osteoprotegerin (OPG), a natural soluble decoy receptor of RANKL, modulates the effect of RANKL and is able to prevent excessive bone resorption in the normal state. RANKL expression is elevated in patients with MM [63, 64]. Denosumab (AMG 162, Amgen Inc., Thousand Oaks, CA) is an investigational fully human mAb with high affinity and specificity for RANKL that mimics the natural bone-protecting actions of OPG [65]. A phase 1 clinical trial in patients with MM (*n* = 25) or breast cancer with bone metastases (*n* = 29) showed that following a single s.c. dose of denosumab (0.1, 0.3, 1.0, or 3.0 mg/kg), levels of urinary and serum N-telopeptide decreased within 1 day, and this decrease lasted through 84 days at the higher denosumab doses [66]. Mean half-lives of denosumab were 33.3 and 46.3 days for the two highest dosages. Larger trials are ongoing to investigate the effect of denosumab for the treatment of cancer-induced bone disease and other bone loss disorders [67].

#### 4.2.2. Targeting the Wnt Inhibitor Dickkopf-1 (DKK-1)

Dickkopf-1 (DKK1), a soluble inhibitor of wingless (Wnt) signaling secreted by MM cells contributes to osteolytic bone disease by inhibiting the differentiation of osteoblasts. The effect of anti-DKK1 mAb on bone metabolism and tumor growth in a SCID-rab system has been evaluated [68]. The implants of control animals showed signs of MM-induced resorption, whereas mice treated with anti-DKK1 antibodies blunted resorption and improved the bone mineral density of the implants. Histologic examination revealed that myelomatous bones of anti-DKK1-treated mice had increased numbers of osteocalcin-expressing osteoblasts and reduced number of multinucleated TRAP-expressing osteoclasts. The bone anabolic effect of anti-DKK1 was associated with reduced MM burden (*P* < .04). Anti-DKK1 also significantly increased BMD of the implanted bone and murine femur in nonmyelomatous SCID-rab mice, suggesting that DKK1 is physiologically an important regulator of bone remodeling in adults. Anti-DKK1 agents including BHQ880 (Novartis) may therefore represent the next generation of therapeutic options for the enhancement of bone repair in some malignant and degenerative bone diseases including MM [69, 70]. Although BHQ880 had no direct effect on MM cell growth, BHQ880 increased osteoblast differentiation, neutralized the negative effect of MM cells on osteoblastogenesis, and reduced IL-6 secretion. Furthermore, in a SCID-hu murine model of human MM, BHQ880 treatment led to a significant increase in osteoblast number, serum human osteocalcin level, and trabecular bone. Preliminary results from a phase I/II trial in MM where BHQ880 was given IV for 28 days was well tolerated when given in combination with zoledronic acid.

#### 4.2.3. Targeting the Activin Receptor Type IIA (ActRIIA)

ACE-011, a novel bone anabolic agent currently in a Phase 2 clinical trial in MM, is a protein therapeutic based on the activin receptor IIA. In numerous preclinical models of bone loss, ACE-011 has demonstrated beneficial effects on both trabecular and cortical bone [71, 72]. ACE-011 increased bone mineral density, improved bone architecture, increased the mineral apposition and bone formation rates, and improved bone mechanical strength [73]. Results of the Phase 1 study in postmenopausal women demonstrated that a single dose of ACE-011 caused a rapid, sustained, dose-dependent increase in serum levels of bone-specific alkaline phosphatase (BSAP), a marker of bone formation, while a marker of bone resorption, C-terminal type 1 collagen telopeptide (CTX), decreased. In MM an ongoing multicenter Phase 2 trial is conducted in Russian patients which are treated with melphalan, prednisone, and thalidomide and randomized to receive either monthly doses of ACE-011 or placebo for up to three months. Preliminary results show clinical significant increases in biomarkers of bone formation, improvement in skeletal metastases, decreases in bone pain as well as antitumor activity [54]. In summary, these data indicate that ACE-011 is well tolerated and has significant hematologic activity in MM patients receiving myelosuppressive chemotherapy. 

Moreover, ACE-011 has potential as a novel therapy for chemotherapy-induced anemia and may be an effective alternative to erythropoietin- (EPO-) based treatments.

### 4.3. Targeting Angiogenesis by VEGF Inhibitor Bevacizumab (Avastin)

Vascular endothelial factor (VEGF) is important for the formation of new blood vessels and plays a key role not only in solid tumors but also in hematologic malignancies, including MM [74]. Bevacizumab targets and blocks VEGF and VEGF's binding to its receptor on the vascular endothelium [75]. Anti-VEGF Abs were active alone, and in combination with radiation in earlier preclinical studies [75, 76]. It is currently being studied clinically in many solid and blood tumors including primary systemic amyloidosis and MM [77, 78]. NCI's Cancer Therapy Evaluation Program is sponsoring a phase II study of bevacizumab plus thalidomide in MM [78].

### 4.4. Targeting BAFF/ARPIL Growth and Survival Pathway by Atacicept (TACI-Ig) or BAFF Inhibitor

Recently, B-cell activating factor of the tumor necrosis factor (TNF) family (BAFF; also known as B lymphocyte stimulator, BLyS) and a proliferation inducing ligand (APRIL), were identified as new survival factors for MM [79–81]. In addition to BMSCs, osteoclasts produce these factors to support MM cells in the BM microenvironment [81, 82]. Their cognate receptors are BAFF-R/BR3, transmembrane activator and calcium modulator (TACI), and B-cell maturation antigen (BCMA) with heterogeneous expression among patient MM cells. Specifically, RNA expression of BCMA and TACI is approximately >30-fold and >10-fold higher, respectively, than that of BR3 [81]. BR3 specifically binds BAFF but not APRIL, and has very limited expression in mature B-cells plasma cells [83]. In fact, BCMA expression is only acquired in mature B cells and accompanied by loss of BAFF-R expression [83], suggesting a key role of BCMA in plasma cell survival. These studies provide clinical rationale to target BAFF/APRIL survival pathway in MM. 

Atacicept (TACI-Ig, ZymoGenetics; Serono) acts as a decoy receptor by binding to and neutralizing soluble BAFF and APRIL, and preventing these ligands from binding to their cognate receptors on B-cell tumors, thereby enhancing cytotoxicity. An open-label, dose-escalation Phase I/II study enrolled 16 patients with refractory or relapsed MM (*n* = 12) or active, progressive Waldenstrom's Macroglobulinemia (*n* = 4) [84]. Atacicept was well tolerated and showed clinical and biological activity consistent with its mechanism of action. TACI was expressed heterogeneously among patient MM cells, which may explain promising results for the treatment of TACI^high^ MM cells in a trial for atacicept [84, 85].

In addition, the *in vivo* antitumor activity of neutralizing anti-BAFF mAb in SCID-hu model of human MM provide the preclinical rationale for its evaluation in the treatment of MM [86]. Moreover, since all MM cell lines and patient MM cells express BCMA, BCMA might be a promising target for monoclonal antibody development against MM. Importantly, MM cells in remission postallogeneic transplant due to graft-versus-tumor response have donor derived anti-BCMA Abs that are tumor lytic *in vivo* [87]. Thus, BCMA is a target of donor B-cell immunity in patients with myeloma who respond to donor lymphocyte infusion (DLI). Antibody responses to cell-surface BCMA may contribute directly to tumor rejection *in vivo*. Indeed, BCMA antibodies show cytotoxic activity both as naked IgG and as drug conjugates, which warrant further evaluation as therapeutic candidates for plasma cell malignancies [88].

### 4.5. Other Potential Targets

Additional mAbs are directed against a variety of further MM cell targets including HLA-DR by 1D09C3 [89], HLA-class I by 2D7-DB [90], kininogen by C11C1 [91], and polyclonal rabbit antithymocyte globulin (rATG) [92].

Finally, since NK cells play a critical role in ADCC to lyse tumor target cells via therapeutic monoclonal antibodies and inhibitory-cell killer immunoglobulin-like receptors (KIRs) negatively regulate natural killer (NK) cell-mediated killing of HLA class I-expressing tumors, mAbs targeting KIR might prevent their inhibitory signaling leading to enhanced ADCC. A novel fully human anti-KIR blocking mAb, 1-7F9 (or IPH 2101), antagonizes inhibitory KIR signaling, activates NK cells and augments natural killer-mediated killing of tumor cells [93, 94]. Importantly, 1-7F9 enhances patient NK cell cytotoxicity against autologous MM tumor cells *in vitro* and appears safe in an ongoing phase I clinical trial [95]. A multicenter, open label Phase IIa clinical trial (trial IPH 2101-201, in France) has started to evaluate IPH 2101 as a single agent in patients with stable measurable MM after induction therapy. Another phase II clinical trial to assess the potential of lenalidomide combined with 1-7F9 will be initiated in patients with MM.

## 5. Conclusion

For the past decade, more than a dozen of therapeutic mAbs have either entered clinical trials or in clinical development in MM. However, mAbs targeting myeloma cells have not yet been included as part of standard myeloma therapy. Although the ability to create essentially human antibody structures has reduced the likelihood of host-protective immune responses that otherwise limit the utility of therapy, majority of MM patients are immunosuppressive. The immediate goal would be testing next generations of genetically Fc-engineered mAbs that not only binds to target MM antigens with high affinity but also have superior interaction (>1 log) with host immune effectors. A better understanding of the immune defects that prevent MM patients from mounting a strong response against their tumor cells should also improve establishment of effective mAb-based immunotherapy strategies for MM. We expect that, the use of potentially targeted therapies by mAbs, such as Fc-engineered naked or immunoconjugate or bispecific would soon claim defined therapeutic roles in patients with MM. The favorable toxicity profile of tumor-targeted therapy by mAbs, unlike other forms of therapy, may allow the maintenance of quality of life, while efficiently attack the tumors.

## Figures and Tables

**Table 1:** Antigens targeted by antibodies in multiple myeloma in different stages of preclinical/clinical development.

Target	Brand name	Company/Sponsor	Type of mAb (conjugate)	Phase	Remarks
CD138	B-B4-DM1	ImmunoGen	the maytansinoid immunoconjugate mouse IgG1 mAb B-B4	preclinical	Tassone Blood 2004,104:3688–96

HM1.24	humanized HM1.24	Chugai Pharmaceutical	humanized	preclincial	Ozaki Blood 1999,93:3922–3930

	humanized HM1.24	Xencor Inc	Fc-engineered humanized IgG_1_	preclincial	

IL-6	OP-R003-1, 1339 Elsilimomab, Azintrel	OPi EUSA Pharma; Vaccinex licensed to Glaxo Smith Kline	human IgG1	preclincial	Fulciniti Clin Cancer Res 2009,15:7144–52

HLA-DR	1D09C3	GPC Biotech, AG	human IgG1	preclincial	Carlo-Stella Cancer Res 2007

kininogen	C11C1	Temple University School of Medicine	mouse	preclincial	Sainz Cancer Immunol Immunother 2006 C11C1 mAb inhibits its own tumor growth *in vivo*, slows down B38-MM growth rate when both MM are implanted together and when mAb C11C1 is injected intraperitoneally. MAb C11C1-treated-MM showed decreased MVD and kininogen binding *in vivo* without FGF-2, B1R, or B2R expression changes

HLA class I	2D7-DB	Chugai Pharmaceutical Co. Ltd.	converted from mouse IgG2b, single-chain Fv diabody	preclincial	Sekimoto Cancer Res 2007; 67:1184–92. a recombinant single-chain Fv diabody 2D7-DB specifically induces multiple myeloma cell death in the bone marrow environment

*β*2-microglobulin	anti-*β*2M mAbs	MD Anderson Cancer Center	mouse	preclincial	Yang Blood 2007; 110:3028–35. & Clin Cancer Res 2009; 15:951–9. Strong apoptotic effect on myeloma cells and low toxocity in the mice suppports potential use as therapeutic agents

CD38	MOR202	MorphoSys AG	human IgG1	preclincial	Tesar et al. J. Clin Oncol 2007, 25(18S): 8106

CD32B	MGA321(2B6)	MacroGenics	humanized IgG1	preclincial	Zhou Blood 2008; 111:549–557. humanized 2B6 MoAb may target in patients with systemic AL-amyloidosis. It blocks Fc engagement of CD32B and may improve the performance of other cancer Mabs when combined with them during administration

FGFR3	PRO-001	Prochon Biotech Ltd.	human IgG1	preclincial	Trudel Blood 2006; 2:4908–4915. The inhibitory anti-FGFR3 antibody, PRO-001, is cytotoxic to t(4;14) MM cells and deserves further study for the treatment of FGFR3-expressing myeloma

ICAM-1	cUV3	Abiogen	chimeric IgG1	preclincial	Smallshaw J Immunother 2004; Coleman J Immunother 2006 cUV3 significantly prolongs the survival of SCID/ARH-77 mice

BLyS	BLyS/rGel	Targa Therapeutics	Fusion protein of an antibody tethered to a toxin	preclincial	Lyu et al. Mol Cancer Ther 2007; 6:460–70

TACI	Atacicept (TACI-Ig)	ZymoGenetics Inc.	fusion protein	preclincial	Yaccoby Leukemia 2008 22, 406–413

CD70	SGN-70	Seatle Genetics	humanized IgG1	preclincial	McEarchern Clin Cancer Res 2008 14, 7763–72

TRAIL-R2(DR5)	lexatumumab	Human Genome Sciences	human	preclincial	Menoret et al. Blood 2006;132: 1356–62

IL-6R	NRI (engineered Tocilizumab)	Roche Pharmaceuticals	a single-chain fragment format dimerized by fusing to the Fc portion of human immunoglobulin G1	preclincial	Yoshio-Hoshino Cancer Res 2007; 67:871–5. the NRI gene introduction combined with adenovirus gene delivery inhibited the *in vivo* S6B45 cell growth significantly

BCMA	SG1	Seatle Genetics	Auristatin-BCMA mAb	preclincial	Ryan et al. Mol Cancer Ther 2007; 6:3009–18

matriptase	M24-DOX	UMDNJ—The Cancer Inst. of New Jersey	immunoconjugate with doxorubicin	preclincial	Bertino et al. 2010 AACR abtract no. 2596. M24-DOX is as potent as free doxorubicin to inhibit the growth of MM cells. But target delivery of doxorubicin by the matriptase antibody significantly reduced the toxicity toward cardiomyocytes that lack matriptase expression

IL-1beta	XOMA 052	XOMA (US) LLC	Human Engineered IgG2	preclincial	Lust 2010 AACR abstract no. 2449. XOMA 052 is highly effective at inhibiting IL-1 induced IL-6 production in myeloma patients *in vitro *

CD20	Rituxan	NCI & Memorial Sloan-Kettering Cancer Center	chimeric with a human IgG1 Fc	II (ongoing)	NCT00258206 (with cyclophosphamide): NCT00505895. High-dose cyclophosphamide in combination with rituximab in patients with primary refractory, high-risk, or relapsed myeloma, also being studied for the treatment of peripheral neuropathy in patients with MGUS

CD20	Zevalin (yttrium Y 90 ibritumomab tiuxetan)	NCI	mouse IgG1	I (ongoing)	NCT00477815: Zevalin radioimmunotherapy with high-dose melphalan and stem cell transplant for MM

CD40	SGN-40 (Dacetuzumab)	Seatle Genetics/Genentech	humanized IgG1	I b (ongoing)	NCT00664898: safety and pharmacology of SGN-40 administered in combination with Bortezomib (Velcade, PS-341) in patients with relapsed or refractory MM. NCT00525447 is the study of SGN40, lenalidomide, and dex in MM patients

CD40	HCD122 (Lucatumumab)	Novartis	human IgG1	I (ongoing)	NCT00231166 Dose-finding trial of HCD122 in MM patients that is relapsed or has not responded to prior therapy

CD20	Bexxar (131-tositumomab)	GlaxoSmithKline	radioactive iodine 131 attaching to anti-CD20; muIgG2a (131)	II (ongoing)	NCT00135200: to see whether the treatment with Bexxar will decrease and possibly eliminate residual myeloma cells resistant to chemotherapy

CD56	BB-10901 (IMGN901)	ImmunoGen, Inc.	humanized (maytansine DM1 conjugation)	I (ongoing)	NCT00346255: given as an intravenous infusion weekly for two consecutive weeks every three weeks to relapsed and relapsed refractory CD56-positive MM; NCT00991562: IMGN901 in combination with lenalidomide and dexamethasone

RANKL	Denosumab	Amgen	human IgG2	II/III (ongoing)	NCT00259740: to determine if denosumab is effective in the treatment of relapsed or plateau-phase MM; NCT00104650: to determine the effectiveness of AMG 162 in reducing urinary N-telopeptide in advanced cancer subjects with bone metastases; NCT00330759: Phase III Study of Denosumab Compared With Zoledronic Acid (Zometa) in the Treatment of Bone Metastases in Subjects With Advanced Cancer (Excluding Breast and Prostate Cancer) or MM

VEGF	Avastin beuacizumab	Genentech	humanized	II (ongoing)	NCT00428545 (in combination with bortezomib); NCT00410605 (added with lenalidomide and dexamethasone)

CD52	Campath-1H (alemtuzumab)	NCI; Fred Hutchinson Cancer Research Institute	humanized	II (ongoing)	NCT00625144: studying the side effects of giving fludarabine and busulfan together with alemtuzumab followed by donor stem cell transplant and to see how well it works in treating patients with hematological cancer or other disease

IL-6	CNTO 328	Centocor, Inc	chimerized IgG1	I/II (ongoing)	NCT00401843 (in combination with bortezomib); NCT00911859 (added with Velcade-Melphalan-Prednisone); NCT00402181 (in combination with dexamethason)

IL-6	B-E8 (Elsilimomab)	Orphan Pharma International and Diaclone SA	murine	II	Preliminary efficacy was seen but there is a limitation for the clinical use of a murine monoclonal antibody since it frequently induces human anti-mouse antibodies (HAMA)

IL-6R	MRA (Tocilizumab)	Roche Pharmaceuticals	humanized	II	

TRAIL-R1(DR4)	Mapatumumab(TRM-1)	Human Genome Sciences	human	II (ongoing)	NCT00315757 (in combination with bortezomib)

EGFR	Erbitux(EMMA-1)	Imclone; Bristol Meyers-Squibb	chimerized	II (ongoing)	NCT00368121 (in combination with dexamethasone)

CS1	elotuzumab/HuLuc63	Facet Biotech; Bristol-Myers Squibb	humanized	I /II (ongoing)	NCT00742560 & NCT00726869 (in combination with bortezomib)

CD38	HuMax-CD38	Genmab	human IgG1	I/II (ongoing)	NCT00574288: to establish safety profile of HuMax-CD38, given as monotherapy in patients with MM relapsed or refractory to at least 2 different cytoreductive therapies and without further established treatment options

CD38	SAR650984	Sanofi-Aventis; ImmunoGen	humanized IgG1	I (ongoing)	NCT01084252: to determine the maximum tolerated dose (MTD)/maximum administered dose (MAD)

DKK	BHQ880	Novartis	human IgG1	I/II (ongoing)	NCT00741377: in combination with Zoledronic Acid in relapsed/refractory myeloma

CD138	BT062	Biotest; ImmunoGen	chimeric (B-B4-maytansinoid DM4)	I (ongoing)	NCT00723359

the activin receptor type IIA (ActRIIA)	ACE-011	Acceleron Pharma, Inc	human IgG1	I/IIa (ongoing)	NCT00747123 (in patients with osteolytic lesions with MM)

IGF-1R	AVE1642	Sanofi-Aventis	humanized	I/II (ongoing)	Descamps et al. (B J Cancer 2009; 100:366) Anti-IGF-1R Monoclonal Antibody combined with bortezomib for patients with rel/ref MM

Ganglioside GM2	BIW-8962	BioWa, Incorporated	humanized	I/II (ongoing)	Dosing study of anti-GM-2 ganglioside (expressed at high levels on the surface of MM cells) followed by efficacy study

CD74 (variant MHC II)	milatuzumab (hLL1, IMMU-110)	Immunomedics, Inc.	humanized IgG1 or humanized IgG1 doxorubicin conjugate	I/II (ongoing)	NCT00421525: in patients with recurrent or refractory multiple myeloma who have failed at least two prior standard systemic treatments. Its isotope, drug, and toxin conjugates have high antitumor activity in non-Hodgkin's lymphoma and multiple myeloma *in vitro* and in tumor xenograft models. Stein et al. 2007 & 2009

Alpha-4 integrin	natalizumab (Tysabri)	Biogen Idec	humanized IgG4	I/II (ongoing)	NCT00675428: patients with relapsed or refractory multiple myeloma

MHC II (HLA-DR)	1D09C3	GPC Biotech	human IgG4	I	Carlo-Stella et al. 2007 showed that IFN-gamma-induced up-regulation of HLA-DR results in a potent enhancement of the *in vivo* antimyeloma activity of 1D09C3 in mice. Initial clinical testing with 1D09C3 has not raised any unexpected or unacceptable safety concerns and the maximum tolerated dose has not yet been reached. GPC Biotech has decided to not put further internal resources into developing 1D09C3 due to potential swapping of IgG4 antibody one half of its Y-shaped structure with the half of a different antibody, thus resulting in a new molecule whose properties are unknown. However, the Company will seek a partner for the intellectual property relating to this program

IGF-1R	CP-751,871/figitumumab	Pfizer	human IgG2	I	Lacy et al. (J. Clin Onclo 26:3196) reported that CP-751,871 is well tolerated and may constitute a novel agent in the treatment of multiple myeloma

KIR	IPH 2101	Innate Pharma	human IgG4	I/IIa (ongoing)	NCT00552396 (ASCO May 30 2009 abstract 09-AB-3032) safety and tolerability study for patients with relapsed/refractory MM. Preclinical characterization of 1-7F9, a novel human anti-KIR therapeutic antibody that augments NK-mediated killing of tumor cells (Romagne et al. 2009)

Every effort has been made to obtain reliable data from multiple sources including http://clinicaltrials.gov/, company, and other web sites, but accuracy cannot be guaranteed.
